# Disease Burden of the Kidney Disabled in Korea, 2009–2013: The Gap with That of the Non-Kidney Disabled Continues

**DOI:** 10.3390/ijerph19010249

**Published:** 2021-12-27

**Authors:** Sun-Mi Shin, Hee-Woo Lee

**Affiliations:** 1 Department of Nursing, Joongbu University, 201 Daehak-ro, Chubu-myeon, Chungnam, Geumsan-gun 32713, Korea; healthteam@joongbu.ac.kr; 2 Hemodialysis Unit, Lee Hee Woo Internal Medicine Clinic, 1402 Gyebaek-ro, Seogu, Daejeon 35400, Korea

**Keywords:** disease burden, kidney failure, the kidney disabled, out-of-pocket expenditure, the number of chronic diseases, quality of life

## Abstract

Kidney disability due to kidney failure could be considered to be the most severe of all the internal-organ disabilities. The purpose of this study was to identify the disease burden between the kidney and non-kidney disabled among the internal-organ disabled, based on the number of chronic diseases, annual out-of-pocket expenditure, and quality of life. From 2009 to 2013, 308 people (6.5%) with internal-organ disabilities were extracted out of 4732 people with disabilities in the Korea Health Panel. We compared the disease burden of 136 people with kidney disability (44.2%) and 172 people with non-kidney disability (55.8%), and confirmed the trend of disease burden over five years through panel analysis. The disease burden gap between kidney and non-kidney disabilities was, respectively, the number of chronic diseases (4.7 vs. 3.3, *p* < 0.0001), annual out-of-pocket expenditure ($1292 vs. $847, *p* < 0.004), and quality of life score out of 100 (49.2 vs. 60.2, *p* < 0.0001). In addition, when looking at the five-year trend of the three disease burden indexes, the kidney disabled were consistently worse than the non-kidney disabled (*p* < 0.01). In conclusion, health policy planners aiming for health equity need to seek practical strategies to reduce the gap in the disease burden among people with disabilities.

## 1. Introduction

Disease burden is the impact of a health problem on a particular population and can be measured using a variety of indicators such as mortality, morbidity, and financial costs. It can facilitate comparing different burdens of health problems and predict future health care needs [1]. Comparing the disease burden of each chronic disease is important because it is used for a variety of purposes, from prioritizing health policies to evaluating policies. In general, kidney failure has a high cost and a high burden of the disease requiring long-term medical services. However, there are few studies that quantitatively compared it with other chronic diseases.

Kidney failure is a stage 5 chronic kidney disease (CKD). Kidney failure is a serious, chronic disease that requires long-term treatment with expensive medical care. Kidney replacement therapy (KRT), including peritoneal dialysis (PD), hemodialysis (HD), or kidney transplantation (KT), is essential in the treatment of kidney failure. Kidney failure rates are increasing in developing countries despite reports that they are stabilizing in developed countries [2]. In 2013, the prevalence of CKD in Koreans was very high: 4.1% in adults over 30 years of age, and 16.5% in adults over 65 years of age [3]. In addition, the recent increase in dialysis patients is 8% to 9% per year [4]. The major contributing factors to kidney failure are diabetes, hypertension, and aging [5,6,7]. Korea became an aged society in 2017, when the population aged 65 and over was 14% or more. It is expected to enter a super-aged society in 2026, with 21% or more of the population over 65 years old [8].

Compared to non-disabled people, disabled people have a higher prevalence of chronic diseases, such as obesity, hypertension, and diabetes, and their quality of life (QOL) is expected to deteriorate further. In particular, kidney failure has high severity among all chronic diseases, so it is possible to hypothesize that the severity of kidney disability based on kidney failure will be higher than that of other persons with disabilities. Therefore, it is necessary to check the disease burden according to the type of disability, and it will be reasonable to intervene in the health and welfare policy considering such disparity.

There are various classifications of people with disabilities, such as medical classification and functional classification [9]. In Korea, disability types are classified according to the medical model, and they can be divided into external disabilities, internal-organ disabilities, and mental and developmental disabilities [10]. Among these, internal-organ refers to disabilities that affect one or more of various internal-organ of the body, such as the liver, heart, and kidneys [11]. Many of these internal medical conditions are progressive diseases that lead to long-term illness and require ongoing medical and welfare services. Among these, people with kidney disabilities are those who have undergone dialysis or a kidney transplant because of kidney failure. They are each officially registered to the government as a kidney disability [11].

Compared to the general public, registered disabled people maybe receive exemptions from mandatory health insurance or various medical welfare benefits from the government in Korea. For example, there are social welfare services such as basic living expenditure support, free transportation service, and information-communication cost support. In addition, registered disabled persons with low income and low assets are designated as Medical Aid (MA) with free or very low copayments when using medical services. Since MA is operated by national tax, the government selects beneficiaries through the government’s strict eligibility screening process. As of 2020, only about 3% of all Koreans are eligible for MA, and they are among the poorest [12,13]. However, about 30% of registered disabled people are receiving Medical Aid (MA). This is a result of reflecting the implementation of the MA system for the medical welfare of the poor, as the disabled can fall into poverty more than the general population. As the number of registered disabled people increases, the cost of medical benefits based on tax increases, which becomes a financial burden on the government and the people.

This study was designed based on the hypothesis that the disease burden of kidney failure among all chronic diseases would be high and that the disease burden of the kidney disabled would be higher than that of the non-kidney disabled. Therefore, the purpose of this study is to illuminate the burden of kidney disabled people using the Korea Health Panel (KHP) data, which is one of the representative data of Korea. In this case, the disease burden was calculated using three variables, the number of chronic diseases, the annual out-of-pocket (OOP) medical expenditure, and today’s subjective health status as QOL (EQ visual analog scale, EQ-VAS) [14]. In the future, the results of this study can be used as basic data for various medical welfare policy measures to overcome the relatively more serious disease burden of the kidney failure. It is also expected to provide implications for the severity of kidney failure among all serious internal-organ diseases.

## 2. Materials and Methods

### 2.1. Data Source

The KHP is one of the nationally recognized representative data, and it is used to prepare the basis for medical policy reform by collecting data related to the health level and medical use of Koreans [15]. The basic survey was started in 2008, and additional surveys on health behavior and QOL were started in 2009, and the survey has been continued until now [16]. Subjects were selected by stratified multistage sampling and the same or slightly modified variables are collected every year, but new variables are sometimes surveyed due to new health issues [17]. We received data for seven years from 2008 to 2014 from Korea Institute for Health and Social Affairs (KIHASA) for this study in 2016, but QOL, the main target variable of this study, was investigated from 2009. In addition, the data for 2014 was a beta version that had not yet secured stability from KIHASA. Therefore, this study used five years of data from 2009 to 2013 [18]. It was officially approved by the institutional review committee (KIHASA 2016-01).

### 2.2. Data Collection

The KHP questionnaire consisted of households and family members, and additional surveys such as health behavior and QOL were separately conducted only for adults nineteen years of age or older. All variables were surveyed by an experienced investigator visiting the family. In order to investigate the exact date, the reason for medical use, and OOP medical expenditure, etc., the National Health Insurance (NHI) Corporation and KIHASA made a standardized medical use account book and educated the subjects on how to record it in advance. In addition, the subject kept the receipt together with the faithful record of the medical use status before forgetting. In addition, during the survey, the investigator compared and reviewed the subject’s medical use record and health insurance claim data, making every effort to ensure that there were no omissions or erroneous records [17].

### 2.3. Study Subjects and Analytic Variables 

In this study, three target variables were used to measure disease burden: the number of chronic diseases diagnosed by physicians, annual OOP medical expenditure, and today’s subjective health status as QOL (EQ-VAS). Explanatory variables or covariates include type of disability, age, gender, marital status, economic activity, and type of medical social security. 

The subjects of this study were 308 persons with internal-organ disabilities among panel subjects for five years, from 2009 to 2013. These correspond to 6.5% of the 4732 persons with all kinds of disabilities in the KHP data during the same period. In order to confirm the clear difference between kidney and non-kidney disabilities, we classified internal-organ disability as kidney and non-kidney disability (heart, respiratory, liver, colostomy or urostomy, epilepsy). This is because internal disabilities are rare among people with disabilities. Among them, kidney disability (*n* = 136) consisted of dialysis (*n* = 115) and KT (*n* = 21). Non-kidney disability (*n* = 172) included disabilities of the heart (*n* = 25), respiratory organ (*n* = 43), liver (*n* = 5), colostomy or urostomy (*n* = 32), and epilepsy (*n* = 67). In addition, looking at five years pooled data by each year, subjects with kidney and non-kidney disabilities were 23 vs. 41 in 2009, 23 vs. 0 in 2010, 30 vs. 43 in 2011, 28 vs. 41 in 2012, and 32 vs. 47 in 2013. 

### 2.4. Statistical Analysis

Socio-demographic characteristics of the subjects were identified through t or chi-square tests using long-format pooled data. The number of chronic diseases, the annual OOP medical expenditure, and today’s subjective health status as QOL points (EQ-VAS) were determined by ANCOVA with covariates such as gender, age, marital status, and economic activity using pooled data. Additionally, this study performed a population weight analysis to overcome the bias caused by the sample data. 

The mixed-effect model of panel analysis after control of all possible confounding variables presented five-year trends of the number of chronic diseases, the annual OOP medical expenditure, and today’s subjective health status as QOL (EQ-VAS) with a locally weighted scatterplot smoother (LOWESS) curve. At this time, the annual OOP medical expenditure (Korean Won, KRW) was converted to US Dollars ($) as of 1 July 2009 (1 $ = 1258.59 KRW) [19]. Missing data were excluded from all analyzes using SAS 9.4 (SAS Institute, Cary, NC, USA), and only *p*-values less than 0.05 were considered statistically significant.

### 2.5. Definitions of Terms

#### 2.5.1. Disease Burden

In this study, three indicators were selected to measure the disease burden: the number of chronic diseases, the annual OOP medical expenditure, and today’s subjective health status as QOL (EQ-VAS).

The number of chronic diseases refers to the number of comorbidities that the subject suffers from, and is recognized when the subject has been diagnosed with hypertension, hyperlipidemia, cerebrovascular disease, ischemic heart disease, diabetes, tuberculosis, etc. [16]. The KHP data are managed by entering the investigated chronic disease as a standardized disease code (KCD6_CODE) [20].

The annual OOP medical expenditure refers to the sum of non-reimbursement and the medical expenditure that should copay after excluding insurance benefits of visiting the emergency room, admission ward, outpatient department (OPD), and purchasing pre-scription drugs. Therefore, these were included in the OOP medical expenditure for each person with internal-organ disabilities, such as cost for dialysis or cost for KT. So far, research on medical expenditure has analyzed each insurance claim data, but there have been few studies that measured individual copayments as in this study.

Today’s subjective health status as QOL (EQ-VAS) is a measure of how many points the subject himself or herself had on a vertical scale based on the lowest 0 and highest 100 points of health status on the day of the interview [14]. A limitation of the visual analog scale is the end-aversion bias, which states that respondents are less likely to use both extremes of the scale. However, it is still a useful method because it is a simple and intuitive method among various tools to evaluate health-related QOL [21].

#### 2.5.2. The Internal-Organ Disabled, Kidney or Non-Kidney Disabled

The internal-organ disabled means people with organ disabilities such as the kidney, heart, liver, respiratory organ, colostomy or urostomy, and epilepsy. People with internal organ disorders find it difficult to maintain stable economic conditions due to severe health conditions and long-term treatment costs. As a result, they receive various welfare benefits by becoming a registered disabled person according to the Welfare Act for the Disabled [22]. 

In this study, although the number of persons with internal-organ disabilities was not large despite the five-year pooled data, the study subjects were classified into those with kidney disabilities and those the non-kidney disabled. People with kidney disabilities are particularly those on KRT therapy for kidney failure. Other people with internal-organ disabilities were named non-kidney disabled.

#### 2.5.3. Medical Social Security System

The Korean medical social security system consists of the NHI and public assistance MA. NHI is a mandatory system of the social security network. Koreans enrolled in health insurance are required to pay premiums to the NHI Corporation according to their income and asset level. 97% of the people are obligated to join the National Health Insurance. Their copayments range from 20% up to 60% of the total cost for each medical service [23]. 

Meanwhile, MA supports the lowest income group or the persons with an incapacity for maintaining their life. They are covered by a tax-based MA system without paying obligatory NHI premiums. Their admission fees are either waived or significantly lower than NHI (0–10% of total cost), and OPD costs are also small (0.95–15% of total cost) [23]. In Korea, about only 3% of the people receive MA [12,13]. People with disabilities are more likely to fall into poverty because they are generally more vulnerable and need more health care [24,25]. People with disabilities receive a variety of social services from the government, but only about 30% of them receive MA. Therefore, it can be predicted that the economic hardship of the kidney disabled person, who is expected to have relatively catastrophic health expenditure, will be greater. In order for a person with kidney failure to be registered as kidney disabled, they must have been receiving dialysis treatment for at least three months immediately before the disability registration, and the doctor of the relevant medical institution must confirm this. In addition, in order for persons with disabilities to be enrolled in MA, they must be selected after reviewing household income, the ability of dependents, and working ability.

## 3. Results

### 3.1. Socio-Demographic Characteristics

Out of 308 persons with internal disabilities for five years, 136 persons with kidney disabilities and 172 persons with non-kidney disabilities were identified. The mean age of the kidney disabled and non-kidney disabled was 59.5 and 56.5 years old (*p* < 0.003) and the proportion of males were also different, 47.8% and 72.7% (*p* < 0.0001), respectively. In the five-year KHP pooled data, kidney disability consisted of dialysis (*n* = 115) and KT patients (*n* = 21), and non-kidney disability included disabilities of the heart (*n* = 25), respiratory organ (*n* = 43), liver (*n* = 5), and colostomy or urostomy (*n* = 32), and epilepsy (*n* = 67). 

In a population-weighted analysis, the number of kidney disabled was 335,180 in the five-year data, which was equivalent to 134 per million. The statistical significance in subjects with population weights was the same as the study subject (Table 1).

### 3.2. The Number of Chronic Diseases

The number of chronic diseases held was 4.7 for the kidney disabled on average, and 3.3 for the non-kidney disabled on average (*p* < 0.0001). Similarly, in the population-weighted analysis, there was a statistical difference in the number of chronic diseases between the kidney and non-kidney disabled (*p* < 0.0001) (Table 2).

### 3.3. The Annual OOP Medical Expenditure

For a year, the annual OOP medical expenditure was about $1292 for the kidney disabled and $847 for the non-kidney disabled (*p* < 0.004). Similarly, in the population-weighted analysis, the statistical significance of the annual OOP medical expenditure for the kidney and non-kidney disabled were the same (Table 3).

### 3.4. Today’s Subjective Health Status as QOL (EQ-VAS)

The result showed that among the best points (100), the average response point for the kidney disabled was 49.2, which was lower than 60.2 for the non-kidney disabled (*p* < 0.0001). Similarly, in the population-weighted analysis, the statistical significance of the QOL of the kidney and non-kidney disabled were the same (Table 4).

### 3.5. The Trends over Five Years of the Number of Chronic Diseases, the OOP Medical Expenditure, and QOL

**Figure 1:**
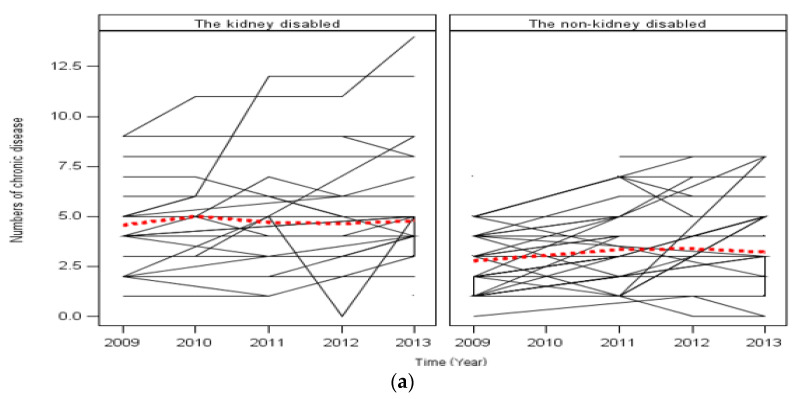

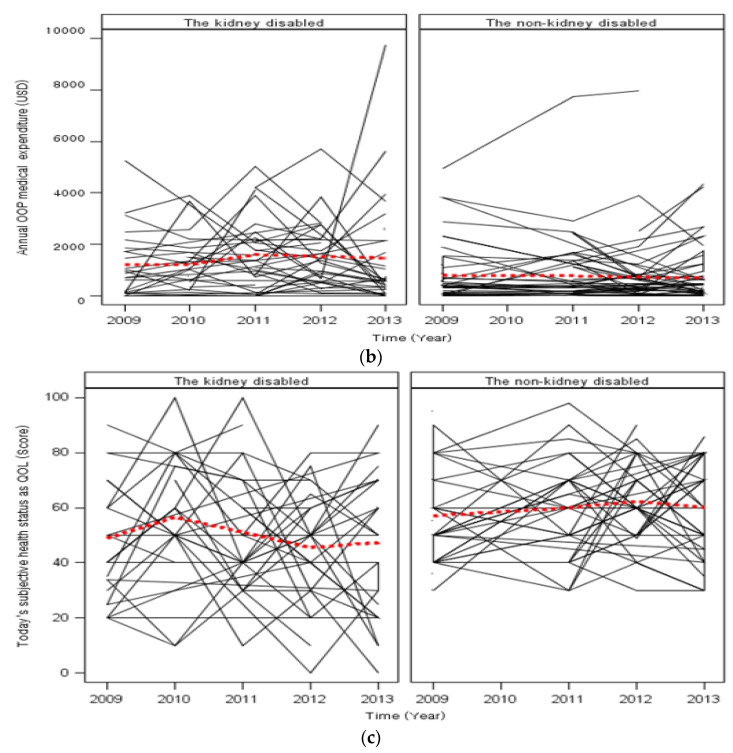
Five years of trends of chronic disease numbers, the OOP medical expenditure, and QOL in both groups by a mixed-effect model using unbalanced panel data. Thick red dotted lines refer to LOWESS curve, and other lines refer to the value of each subject. The covariate variables were age, gender, marital status, economic activity, and type of medical social security; (**a**) There was a difference between the kidney disabled and the non-kidney disabled in the five-year trend in the number of chronic disease (*p* < 0.01); (**b**) There was a difference between the kidney disabled and the non-kidney disabled in the five-year trend in the annual OOP medical expenditure (*p* < 0.01). (**c**) There was a difference in QOL (today’s subjective health status) in the five-year trend between the kidney disabled and the non-kidney disabled (*p* < 0.001).

A panel analysis adjusted for age, gender, marital status, and economic activity revealed trends over five years: the number of chronic diseases, annual OOP medical expenses, and today’s health status as QOL. The results of the kidney disabled were worse than those of the non-kidney disabled throughout five years (*p* < 0.01) (Figure 1). 

## 4. Discussion

Health equity can be reached when the disease burden of each chronic disease is evaluated and health welfare policies are implemented in consideration of this. 

According to previous studies, kidney failure is more serious among all chronic diseases, and the severity of kidney failure increases when accompanied by multiple chronic diseases [26,27,28,29]. Also, the QOL of various types of people with disabilities among the population was relatively lower [29,30]. However, few studies have quantitatively compared the severity of disease and QOL between types of disability. Our research hypothesis is that there is a difference in the disease burden for each type of disability. In particular, it is predicted that the disease burden on the kidney disabled due to kidney failure is greater than that of non-kidney disabled people. Therefore, this study analyzed the demographic characteristics of people with kidney disabilities and compared the disease burden of the kidney disabled with the non-kidney disabled based on the number of chronic diseases, the annual OOP medical expenditure, and QOL.

Among the KHP data for five years (2009~2013), there were 308 persons with internal-organ disabilities. Among them, there were 136 people with kidney disabilities, and when weighted as the population, this became 335,180. Therefore, when the prevalence of kidney disabilities per 1 million Koreans is calculated, it corresponds to 1341 persons. This is less than the 1446 KRT subjects per million Koreans announced in 2013 [4]. This is thought to be because our results were only for the kidney disabled. Therefore, there is a possibility that there are people with kidney failure that have not yet been registered as kidney disabled, and social efforts to discover them will be necessary. In addition, it is necessary to substantially reduce the disease burden by expanding the MA beneficiary for all kidney failures.

In this study, the economic activity of the non-kidney disabled was 33.9%, but the kidney disabled were only 12.5% (*p* < 0.001). This reflects that due to the pathological and physiological characteristics of kidney failure, subjects already suffer from physical, mental, and social crises. Kidney failure subjects have difficulty with full-time activities over long periods [29,31]. In previous Korean studies, only 22% of HD and 29% of PD worked full time [4]. Therefore, it is necessary to strengthen the labor policy that encourages the employment of the disabled and to eliminate negative perceptions of the disabled. In previous studies, despite the complete or partial recovery of kidney function after KT, there is still a question about the necessity of registering the disabled [32]. It may be an issue to be considered in terms of fair allocation of limited welfare resources.

The prevalence and care of two or more multiple chronic diseases are important public health concerns. Chronic disease is a cause of mortality, and medical expenditure goes up when there are multiple comorbidities [33,34]. As a result of this study, the number of chronic diseases was higher in the kidney disabled than in the non-kidney disabled (4.7 vs. 3.3, *p* < 0.001). Our result is after controlling for covariate variables such as gender, age, and economic status. In previous studies, finding out about the number of chronic diseases was rare. However, considering the result of about 25% of US adults with two or more multiple chronic diseases [35], comorbidities with the disabled are more worrisome. In addition, the five-year trends for chronic disease disparity between the kidney and non-kidney disabled were significant (*p* < 0.01) in our study. It is the same as the previous result that kidney failure accompanies many chronic diseases [36]. 

Moreover, in this study, the annual OOP medical expenditure was $1292 in the kidney disabled and $847 in the non-kidney disabled (*p* < 0.004). The trends over five years of annual OOP medical expenditure showed a significant difference between the kidney disabled and the non-kidney disabled (*p* < 0.01). As such, the OOP medical expenditure with kidney disabilities is high among serious internal-organ disabilities, and the gap persists throughout the five years. Therefore, it is necessary to consider vertical equity, which guarantees OOP medical expenditure for people with kidney disabilities more than other people with disabilities. Even in the US, annual OOP spending on kidney failure has been concerned about twice that of Medicare. Subjects with ESRD face higher annual OOP and have higher spending on medication-induced OOP. As a result, kidney failure subjects possibly decrease using medication and could be a gap in follow-up treatment [37].

In this study, QOL also evaluated today’s subjective health status (EQ-VAS) out of 100. As a result, the kidney disabled had 48.9 and the non-kidney disabled had 60.4 (*p* < 0.001). The QOL trend through a five-year panel analysis from 2009 to 2013 was also lower than that of the non-kidney disabled (*p* < 0.01). In previous studies, the QOL of kidney failure was lower in the areas of physical activity and pain/discomfort [38,39], but more research is needed for generalization. 

As we hypothesized, this study was able to find out that people with kidney disabilities had a greater disease burden than all other internal-organ disabled, and could also draw implications that should be considered in health and welfare policies. Nevertheless, the limitations of this study are as follows: first, this study is for the disabled with kidney failure. Therefore, it is difficult to generalize the total kidney failure. Second, since the number of internal-organ disabled was not large, the author grouped the kidney disabled and the non-kidney disabled. Therefore, the different characteristics of each internal-organ disability were not considered. Third, although the two patients are not homogeneous because the patients after kidney transplantation have a better life compared to the dialysis patients [40], it was impossible to analyze the difference between the two because the raw data of KHP already combines a KT with dialysis subjects. Fourth, it did not consider the cost of over-the-count medicine or herbal medicines. Therefore, the OOP medical expenditure in this study may be underestimated. 

Nevertheless, this was a rare study that identifies the socio-demographic characteristics, prevalence, and disease burden of the kidney disabled and non-kidney disabled. In a social context, policy support for patients has a significant impact on health opportunities and available resources [41]. This study can provide useful information for the development of health care policies for the kidney disabled. For example, the public does not know much about kidney failure compared to cancer or cerebrovascular disease, which are perceived as dangerous. In addition, the public does not know more about the disease mechanism and burden of kidney failure than other internal organ disorders such as cancer or heart disease. This is probably because kidney failure is a disease with a relatively low prevalence than other diseases. Contrary to popular belief, however, kidney failure can be considered more severe than any other disease, with the worst QOL, and the most expensive cost of disease. 

Therefore, based on the results of this study and other studies, it is necessary to inform the public about the severe disease burden of kidney failure through publicity and education. It is important to know that kidney failure is a serious disease and it will increase in the future, and prevention and management in the pre-stages of severe kidney failure can lead to a different prognosis of the disease. Reinforcing this intelligence would be the beginning of a national effort. On the other hand, social workers and related experts should provide care services through a full understanding and empathy for the complex disease mechanism, the progression of kidney failure, and the disease burden experienced by the kidney disabled. Policymakers in disability medical care should actively review measures to reduce the inequity of the disabled, especially the gap in OOP medical expenditure. The disease burden on the disabled is different, and vertical equity is necessary to compensate for these differences.

## 5. Conclusions

The evidence-based study was rare, even though kidney disability due to kidney failure could be considered to be the most severe of all the internal-organ disabilities. We found that the kidney disabled suffer more severely than any other internal-organ disabled person, and the burden is greater. This was confirmed through the number of chronic diseases, the OOP medical expenditure, and QOL. Therefore, health care policy planners aiming at health equity will be able to find strategies to reduce the gap in the disease burden among people with disabilities when they recognize this. First, we need to find a strategy to prevent entry into the kidney failure phase. However, if kidney failure has already occurred, the quantity and quality of treatment can no longer be reduced to maintain life. In order to achieve this, it is necessary to strengthen the vertical equity of the kidney-disabled person through the medical welfare policy to reduce the out-of-pocket expenditure and improve the QOL. It is necessary to consider the MA policy for people with kidney failure who are not registered as a kidney disabled person, and alleviate the disease burden on registered kidney disabled people. To better improve the QOL and health of people with kidney failure, it will be important for the government to ensure the access and affordability of KRT, which is an essential treatment. We hope that this study can be used for a variety of purposes, from setting priorities in considering health equity for people with disabilities to evaluating health and welfare policies for people with disabilities.

## Figures and Tables

**Table 1:** Socio-demographic characteristics of the kidney and non-kidney disabled in five-year Korea Health Panel (KHP) pooled data.

Classification		Subjects				Population Weight Subject			
		Total (%)	KD ^(1)^ (%)	NKD ^(2)^ (%)	Chi-Square or t/*p*-Value	Total (%)	KD (%)	NKD (%)	Chi-Square or t/*p*-Value
All		308 (100.0)	136 (100.0)	172 (100.0)		792,566 (100.0)	335,180 (100.0)	457,386 (100.0)	
Age	Mean ± SD	58.0 ± 14.8	59.9 ± 11.5	56.5 ± 16.9	2.10/<0.003	53.8 ± 15.0	57.7 ± 11.0	50.8 ± 16.4	4.05/<0.0001
Gender	Male	190 (61.7)	65 (47.8)	125 (72.7)	19.9/<0.0001	468,562 (59.1)	157,164 (46.9)	311,398 (68.1)	35,946.77/<0.0001
	Female	118 (38.3)	71 (52.2)	47 (27.3)		324,004 (40.9)	178,017 (53.1)	145,987 (31.9)	
Age group	<65	180 (58.4)	82 (60.3)	98 (57.0)	0.34/<0.557	560,237 (70.7)	236,815 (70.7)	323,422 (70.7)	0.315/0.5746
	≥65	128 (41.6)	54 (39.7)	74 (43.0)		232,328 (29.3)	98,365 (29.4)	133,963 (29.3)	
Marital status	Married	191 (62.4)	95 (69.9)	96 (56.4)	12.59/<0.002	445,173 (56.5)	230,984 (68.9)	214,188 (47.3)	70,237.1/<0.0001
	Separated, divorced	77 (25.1)	34 (25.0)	43 (25.2)		185,473 (23.5)	82,810 (24.7)	102,663 (22.7)	
	Unmarried	38 (12.4)	7 (5.2)	31 (18.2)		157,412 (20.0)	21,386 (6.4)	136,026 (30.0)	
Economic activity	Yes	75 (24.4)	17 (12.5)	58 (33.9)	18.82/<0.001	198,145 (25.1)	153,450 (33.7)	44,694 (13.3)	42,618.45/<0.0001
	No	232 (75.6)	119 (87.5)	113 (66.1)		592,440 (74.9)	301,954 (66.3)	290,486 (86.7)	
Medical social security	NHI ^(3)^	208 (67.5)	97 (71.3)	111 (64.5)	1.60/0.2	511,799 (64.6)	285,035 (62.3)	226,764 (67.7)	2407.584/<0.0001
	MA ^(4)^	100 (32.4)	39 (28.7)	61 (35.5)		280,767 (35.4)	172,351 (37.7)	108,416 (32.4)	
Type of disability	Dialysis	115 (37.3)	115 (84.6)	-	308.0/0.0001	275,324 (34.7)	275,324 (34.7)	-	792,566/<0.0001
	KT ^(5)^	21 (6.8)	21 (15.4)	-		59,856 (7.6)	59,856 (7.6)	-	
	Heart	25 (8.1)	-	25 (14.5)		52,140 (6.6)	-	52,140 (6.6)	
	Respiratory organ	43 (13.9)	-	43 (25.0)		74,788 (9.4)	-	74,788 (9.4)	
	Liver	5 (1.6)	-	5 (2.9)		12,157 (1.5)	-	12,157 (1.5)	
	Colo- or urostomy	32 (10.4)	-	32 (18.6)		60,652 (7.7)	-	60,652 (7.7)	
	Epilepsy	67 (21.8)	-	67 (39.0)		257,649 (32.5)	-	257,649 (32.5)	

^(1)^ Kidney disability; ^(2)^ Non-kidney disability; ^(3)^ National Health Insurance; ^(4)^ Medical Aid; ^(5)^ Kidney transplantation.

**Table 2:** Comparison of the number of chronic diseases of the kidney and non-kidney disabled by ANCOVA in five-year KHP pooled data.

Classification		Total	KD ^(1)^	NKD ^(2)^	F/*p*-Value ^(3)^
		Number ± SE	Number ± SE	Number ± SE	
Subjects	All	3.9 ± 0.1	4.7 ± 0.2	3.3 ± 0.2	24.08/<0.0001
	Male	3.9 ± 0.1	4.9 ± 0.3	3.5 ± 0.2	14.84/0.0002
	Female	3.7 ± 0.2	4.1 ± 0.2	3.2 ± 0.3	6.53/0.012
Population weight subject	All	3.7 ± 0.2	5.0 ± 0.2	3.4 ± 0.2	33.57/<0.0001
	Male	3.9 ± 0.2	5.9 ± 0.3	3.5 ± 0.3	30.1/<0.0001
	Female	3.5 ± 0.2	4.0 ± 0.2	3.3 ± 0.2	3.9/<0.05

^(1)^ Kidney disability; ^(2)^ Non-kidney disability; ^(3)^ the covariates were age, marital status, economic activity, type of medical social security, and gender for all subjects.

**Table 3:** The annual out-of-pocket (OOP) medical expenditure ($) ^(1)^ for the kidney and the non-kidney disabled by ANCOVA in five-year KHP pooled data.

Classification		Total	KD ^(2)^	NKD ^(3)^	F/*p*-Value ^(4)^
		Mean ($) ± SE	Mean ($) ± SE	Mean ($) ± SE	
Subjects	All	1045.2 ± 77.0	1292.1 ± 109.5	847.5 ± 97.3	8.57/0.004
	Male	914.9 ± 79.9	1197.1 ± 119.7	763.3 ± 85.8	8.26/0.005
	Female	1254.0 ± 152.8	1443.0 ± 190.2	968.4 ± 237.5	2.27/0.135
Population weight subject	All	946.7 ± 73.0	1252.4 ± 107.4	853.3 ± 98.2	7.125/0.007
	Male	896.9 ± 83.8	1234.0 ± 135.6	861.9 ± 102.9	4.48/0.04
	Female	1018.4 ± 132.9	1262.0 ± 176.8	854.2 ± 206.0	2.14/0.15

^(1)^ Including cost for an emergency room visit, out-patient department visit, admission, and buying for the prescribed drug. Every expenditure was converted to USD ($) based on the exchange rate on 1 July 2009 (1 $ = 1258.59 KRW); ^(2)^ Kidney disability; ^(3)^ Non-kidney disability; ^(4)^ the covariates were age, marital status, economic activity, type of medical social security, and gender for all subjects.

**Table 4:** Comparison of the kidney and the non-kidney disabled QOL (EQ-VAS) ^(1)^ by ANCOVA in five-year KHP pooled data.

Classification		Total	KD ^(2)^	NKD ^(3)^	F/*p*-Value ^(4)^
		Mean ± SE	Mean ± SE	Mean ± SE	
Subjects	All	54.9 ± 1.2	49.2 ± 1.7	60.2 ± 1.6	20.3/<0.0001
	Male	54.3 ± 1.6	46.7 ± 2.5	58.8 ± 1.9	13.7/0.0003
	Female	55.9 ± 1.9	53.0 ± 2.3	60.8 ± 3.1	3.95/0.05
Population weight subject	All	54.2 ± 1.2	47.1 ± 1.8	59.9 ± 1.7	26.0/<0.0001
	Male	53.0 ± 1.7	44.8 ± 2.6	58.6 ± 2.1	15.5/0.0001
	Female	55.5 ± 1.9	51.1 ± 2.4	60.6 ± 3.0	5.85/0.01

^(1)^ Today’s subjective health status (0 worst, 100 best); ^(2)^ Kidney disability; ^(3)^ Non-kidney disability; ^(4)^ the covariates were age, marital status, economic activity, type of medical social security, and gender for all subjects.

## Data Availability

Data sets used during the current study are available from the Agency upon reasonable request. Information on the data can be found at the following sites: https://www.khp.re.kr:444/web/data/data.do (accessed on 15 November 2021).

## References

[1] Health Knowledge Measures of Disease Burden (Event-Based and Time-Based) and Population Attributable Risks Including Identification of Comparison Groups Appropriate to Public Health. https://www.healthknowledge.org.uk/public-health-textbook/research-methods/1a-epidemiology/measures-disease-burden.

[2] Wetmore J.B., Collins A.J. (2016). Global challenges posed by the growth of end-stage renal disease. Ren. Replace. Ther..

[3] Chronic Kidney Disease Complications. https://m.ksn.or.kr/general/disease/?sn=4.

[4] Jin D.C. (2015). Dialysis registries in the world: Korean Dialysis Registry. Kidney Int. Suppl..

[5] Hamrahian S.M., Falkner B. (2016). Hypertension in chronic kidney disease. Hypertens. Basic Res. Clin. Pract..

[6] Narres M., Claessen H., Droste S., Kvitkina T., Koch M., Kuss O., Icks A. (2016). The incidence of end-stage renal disease in the diabetic (compared to the non-diabetic) population: A systematic review. PLoS ONE.

[7] Qi W., Li Q., Gordin D., King G.L. (2018). Preservation of renal function in chronic diabetes by enhancing glomerular glucose metabolism. Mol. Med..

[8] Kim B.K., Chun Y.G., Lee S.H., Park D.J. (2015). Emerging technology and institution of foods for the elderly. Food Sci. Ind..

[9] Rah U.W., Jung H.Y. (2009). Changing Concepts and Classifications of Disablement. J. Korean Med. Assoc..

[10] Badley E.M. (1993). An introduction to the concepts and classifications of the international classification of impairments, disabilities, and handicaps. Disabil. Rehabil..

[11] Ministry of Government Legislation Act on Welfare of Persons with Disabilities. https://www.law.go.kr/%EB%B2%95%EB%A0%B9/%EC%9E%A5%EC%95%A0%EC%9D%B8%EB%B3%B5%EC%A7%80%EB%B2%95.

[12] National Health Insurance Corporation The Governance of Korean Health Care System. https://www.nhis.or.kr/static/html/wbd/g/a/wbdga0401.html.

[13] National Health Insurance Corporation. https://www.nhis.or.kr/static/html/wbd/g/a/wbdga0301.html.

[14] EQ-VAS An Important and Under-Used Element of the EQ-5D. https://www.ohe.org/news/eq-vas-important-and-under-used-element-eq-5d.

[15] Korea Health Panel Study. https://www.khp.re.kr:444/eng/main.do.

[16] Korea Health Panel Study. https://www.khp.re.kr:444/eng/data/board/listview.do?bbsid=105.

[17] Korea Health Panel Study. https://www.khp.re.kr:444/eng/survey/sampling.do.

[18] Korea Health Panel Study. https://www.khp.re.kr:444/eng/data/data.do.

[19] Historic Exchange Rates (South Korean Won). http://www.x-rates.com/historical/?from=KRW&amount=1&date=2017-07-20.

[20] Korean Standard Classification of Diseases. https://www.koicd.kr/kcd/kcd.do.

[21] Whitehead S.J., Ali S. (2010). Health outcomes in economic evaluation: The QALY and utilities. Br. Med. Bull..

[22] Statutes of the Republic of Korea Act on Welfare of Persons with Disabilities. https://www.un.org/development/desa/disabilities/wp-content/uploads/sites/15/2019/11/Korea-Republic-of_The-Welfare-Law-for-Persons-with-Disabilities.pdf.

[23] Korea Insurance Charge Review Association. http://www.hicra.or.kr/sub_asp/04_data06_2.html.

[24] Eide A.H., Mannan H., Khogali M., Van Rooy G., Swartz L., Munthali A., Dyrstad K. (2015). Perceived barriers for accessing health services among individuals with disability in four African countries. PLoS ONE.

[25] DeJong G., Palsbo S.E., Beatty P.W. (2002). The organization and financing of health services for persons with disabilities. Milbank Q..

[26] Van Manen J.G., Korevaar J.C., Dekker F.W., Boeschoten E.W., Bossuyt P.M., Krediet R.T., NECOSAD Study Group (2002). How to adjust for comorbidity in survival studies in ESRD patients: A comparison of different indices. Am. J. Kidney Dis..

[27] Martínez-Castelao A., Górriz J.L., Garcia-López F., López-Revuelta K., De Alvaro F., Cruzado J.M. (2004). Perceived health-related quality of life and comorbidity in diabetic patients starting dialysis (CALVIDIA study). J. Nephrol..

[28] Miskulin D.C., Meyer K.B., Martin A.A., Fink N.E., Coresh J., Powe N.R., Levey A.S. (2003). Comorbidity and its change predict survival in incident dialysis patients. Am. J. Kidney Dis..

[29] Kim S., Nigatu Y., Araya T., Assefa Z., Dereje N. (2021). Health related quality of life (HRQOL) of patients with End Stage Kidney Disease (ESKD) on hemodialysis in Addis Ababa, Ethiopia: A cross-sectional study. BMC Nephrol..

[30] Lintern T.C., Beaumont J.G., Kenealy P.M., Murrell R.C. (2001). Quality of Life (QoL) in severely disabled multiple sclerosis patients: Comparison of three QoL measures using multidimensional scaling. Qual. Life Res..

[31] Hogan A.N., Fox W.R., Roppolo L.P., Suter R.E. (2017). Emergent dialysis and its impact on quality of life in undocumented patents with end-stage renal disease. Ethn. Dis.

[32] McGee J., Jackson N.R., Slakey D.P. (2012). Disability and kidney transplantation in the United States. Clin. Transplant..

[33] Collins A.J., Li S., Gilbertson D.T., Liu J., Chen S.C., Herzog C.A. (2003). Chronic kidney disease and cardiovascular disease in the Medicare population: Management of comorbidities in kidney disease in the 21st century: Anemia and bone disease. Kidney Int..

[34] Beddhu S., Bruns F.J., Saul M., Seddon P., Zeidel M.L. (2000). A simple comorbidity scale predicts clinical outcomes and costs in dialysis patients. Am. J. Med..

[35] Ward B.W., Black L.I. (2016). State and regional prevalence of diagnosed multiple chronic conditions among adults aged ≥ 18 years—United States, 2014. MMWR.

[36] Krishnaswami A., Kiley M.L., Anthony F.F., Chen Y., Chen J., Rajagopal S., Paxton E.W. (2016). Effect of advancing age and multiple chronic conditions on mortality in patients with End-Stage Renal Disease after implantable cardioverter-defibrillator placement. Perm. J..

[37] Patel U.D., Davis M.M. (2006). Falling into the doughnut hole: Drug spending among beneficiaries with end-stage renal disease under Medicare Part D plans. J. Am. Soc. Nephrol..

[38] Higuita-Gutiérrez L.F., Velasco-Castaño J.J., Quiceno J.N.J. (2019). Health-related quality of life in patients with chronic kidney disease in hemodialysis in Medellín (Colombia). Patient Prefer. Adherence.

[39] Kraus M.A., Fluck R.J., Weinhandl E.D., Kansal S., Copland M., Komenda P., Finkelstein F.O. (2016). Intensive Hemodialysis and Health-Related Quality of Life. Am. J. Kidney Dis..

[40] Purnell T.S., Auguste P., Crews D.C., Lamprea-Montealegre J., Olufade T., Greer R., Boulware L.E. (2013). Comparison of life participation activities among adults treated by hemodialysis, peritoneal dialysis, and kidney transplantation: A systematic review. Am. J. Kidney Dis..

[41] Boulware L.E., Mohottige D. (2021). The seen and the unseen: Race and social inequities affecting kidney care. Clin. J. Am. Soc. Nephrol..

